# Stunting and Associated Factors among Under-Five-Age Children in West Guji Zone, Oromia, Ethiopia

**DOI:** 10.1155/2021/8890725

**Published:** 2021-02-04

**Authors:** Eyob Afework, Selamawit Mengesha, Demelash Wachamo

**Affiliations:** ^1^Department of Public Health, College of Medicine and Health Sciences, Hawassa University, Hawassa, Ethiopia; ^2^Department of Public Health, Hawassa College of Health Sciences, Sidama National Regional State, Hawassa, Ethiopia

## Abstract

**Background:**

Stunting is one of the most important public health problems in Ethiopia. It remains a problem of greater magnitude particularly in rural and low-income areas. It reflects chronic nutritional deficiencies and illness that occur during the most critical periods for growth and development in early life. It needs proper intervention to save the future, unless it resulted in diminished cognitive and physical development for the rest of their lives. Therefore, this study aimed to assess the prevalence of stunting and associated factors among under-five children in West Guji Zone, Oromia, Ethiopia.

**Method:**

A community-based cross-sectional study was conducted among 767 under-five children who were included in this study by using a multistage sampling technique in 12 kebeles from 3 selected districts. Data were collected from a mother/caregiver of the child by using a structured pretested questionnaire. Standardized anthropometric measurements were used to measure length, weight, and height of a child. Data were entered into Epi Info software version 3.5.1 and exported to SPSS version 23 for analysis for descriptive and logistic regression models.

**Result:**

The prevalence of stunting was 244 (31.8%) with 95% CI (28.6–35.2) among under-five-age children. The under-five children whose fathers had a polygamous marriage (AOR = 4.92, 95% CI: 3.46, 7.00), being female sex (AOR = 1.74, 95% CI: 1.23, 2.47), having below 4 meal frequencies (AOR = 2.95, 95% CI: 1.56, 5.58), not vaccinated (AOR = 1.75, 95% CI: 1.15, 2.67), and from poor households' wealth status (AOR = 3.03, 95% CI: 1.63, 5.63) and also from severely food insecure household (AOR = 2.92, 95% CI: 1.36, 6.24) were short for their age compared with their counterparts.

**Conclusion:**

Nearly one-third of the under-five children were stunted in the study area which needs intervention on child-feeding practice to avoid sex discrimination in the community. In addition to this health officials in collaboration with other sectors, it is needed to act together to improve enforcement of the law for polygamous marriage, the household's wealth status, and food security for the better health of a child and future.

## 1. Background

Stunting is when a child has a low height for their age, usually due to malnutrition, repeated infections, and poor social stimulation [1]. It indicates a community reveals chronic undernutrition and inadequate nutrients for a prolonged period that begins in the womb [2, 3]. Stunting (chronic undernutrition) is one of the major public health concerns that a significant number of children are suffering from moderate-to-severe forms, and the problem is more prominent in countries that are underdeveloped [4].

The 2018 Global Nutrition Report shows there are 150.8 million (22.2%) children under five years are stunted [5]. In Africa, there are 58.7 million under-five children who are stunted [5]. It accounts for 39 percent of stunted under-five children globally. Five regions of Africa have a stunting prevalence of more than 30% including Eastern Africa which has 36.7% of stunting prevalence [6]. Sub-Saharan Africa remains the region with the highest under-five mortality rate in the world in 2017, 76 deaths/1,000 live births [7], and 34% prevalence of stunting [8].

However, Ethiopia working towards the “*Seqota*” Declaration aims to end hunger and undernutrition and to attain the Sustainable Development Goals (SDGs) target to cut stunting by 40% by 2030 [9] and the WHO in stunting target by 2025 [10]. The prevalence of stunting has decreased considerably, from 51% in 2005 to 37%, wasting decreased from 12% to 7%, and underweight children were from 33% to 21% over these 14 years in 2019 [11]. Stunting is one of the most important public health problems in Ethiopia with a double burden of undernutrition comprising both wasted and stunted under-five children. Most studies indicated that stunting is associated with low socioeconomic status, low educational level of parents, poor water supply and sanitation, and high infectious disease burden [12–14].

The basic and major food source for the study area was “*Enset*,” and there was high population density and polygamous marriage was common in the study area. Furthermore, the study area mainly produces cash crop farming such as coffee and khat which were not used for household consumption. It is also important to target specific intervention on the production and utilization of diversified food. In addition to this, it needs to understand major influencing factors to target and intervein the impact of the stunting. Particularly, in the study area, there was the gap of the study to target the interventions based on the contributing factors such as low educated mothers, polygamous marriage, high family size, low income, food insecurity, male sex child preference, infectious disease, diarrheal disease, and other environmental related factors. Hence, it is very important to see the prevalence of stunting and its determinant factors among under-five-age children to reduce the impact of malnutrition. These study findings can be helpful evidence in planning sound interventions to reduce malnutrition among infant and young children and its determinant factors which can be used for public health officials, clinicians, and health planners to reduce the impacts of malnutrition.

## 2. Methods

### 2.1. Study Setting and Design

This study was conducted in the West Guji Zone, Oromia Region, located in the southern part of Ethiopia. It is located 365 km from the capital Addis Ababa in the main road of Addis Ababa. The zonal administration was composed of a total population of 1,241,797 according to the West Guji Zone health and demographic surveillance department dataset. There are also 10 districts and 144 kebeles in the West Guji Zone. Among this, the number of children under 5 was estimated at 193,845. The main crops produced in the area are cash crop such as coffee, maize, and *teff* which is produced in some parts of the district. *Enset* (false banana) is the staple food, and many households consume it. The study design was a community-based cross-sectional study that was conducted from June 01, 2019, to January 2020.

### 2.2. Study Population and Eligibility Criteria

All 6- to 59-month-aged children were the source of population, while all randomly selected children aged 6–59 months and available on the study period were considered as the study population. Those mothers who were unable to respond due to their seriously ill child were excluded from the study.

### 2.3. Study Variables

*Outcome Variable.* Stunting was determined by computing the height-for-age z-score (HAZ) of less than −2 standard deviations (SDs).

*Exposure Variables.* They were sociodemographic and economic variables, obstetric and other maternal characteristics, child morbidity and vaccination status, child-caring practice, and environmental health characteristics.

### 2.4. Sample Size and Sampling Procedures

The sample size (*n*) was calculated using the following single-population proportion formula based on the assumption of (*p*) 37% of children under 5 are short for their age or stunted (below −2 SD) from the 2019 Ethiopia Mini Demographic and Health Survey (EMDHS) [11], 95% confidence interval (CI) (1.96), 5% margin of error (d), design effect (2), and adding 10% contingency:

(1) $$\begin{array}{c} n={\left(Z_{\left(1-\alpha /2\right)}\right)}^{2}\frac{P\left[1-P\right]}{d^{2}}=\frac{{\left(1.96\right)}^{2}\left(0.52\right)\left(0.48\right)}{{\left(0.05\right)}^{2}}=\left(358.2\times 2\right)=716.4+10\%\left(71.64\right)=788.02\approx 788. \end{array}$$

Therefore, the required sample size was *n* = 788 included in the study.

The selection of the study participants, the sampling frame prepared from the West Guji Zone health and demographic surveillance department dataset, which contains child date of birth (age), kebele, household identification, household head name, marital status, and child name, was used. The sample was proportionally allocated to randomly selected 12 kebeles out of 36 kebeles from 3 selected districts.

### 2.5. Data Collection Tools and Procedures

The data were collected through an interview by pretested and structured questionnaires. Sociodemographic and other determinants were assessed. The questionnaires were translated into the local language and validated before the study time was done outside of the study area, and necessary modifications were done based on the findings. Anthropometric measurements such as HFA z-score/LFA z-score were collected from the selected children using a height measuring board to assess stunting. A survey was carried out by trained data collectors and nurses. Principal investigators and supervisors follow the data collection process and check them for consistency and completeness. Mothers of under-five-year aged children were asked to list out food groups and drinks consumed by their children in the previous 24 hrs before the survey. Mothers of children were asked to count their children's meal frequency in the past 24 hrs. [15]. In order to recall the foods and drinks consumed, and meal frequency in the past 24 hrs, the questionnaires were pretested and validated on 5% of the 6–59-month children before two weeks in the study time outside the study area. Then, some modifications to the sequence and arrangement of multiple answer questionnaires were made. Data collectors were five clinical nurses supervised by one Bachelor of Science (B.Sc.) nurse supervisor and investigators. Training and practical demonstrations on interview techniques and physical measurement procedures were given to data collectors for two consecutive days assessed for competency.

### 2.6. Data Analysis

Data entry, cleaning, and analysis were done by SPSS V. 23. Descriptive analysis including frequency distribution and the percentage was made to determine the prevalence of the stunting, to describe socioeconomic and demographic and other determinants. Bivariate logistic regression analysis was conducted for crude odds ratio (COR), and all factors with a *p* value < 0.25 were the candidate to a multivariable logistic regression to control confounding effects. The Hosmer–Lemeshow goodness-of-fit statistic was used to assess whether the necessary assumptions for the application of multiple logistic regression are fulfilled. Adjusted odds ratios (AORs) with 95% confidence intervals (CIs) were used to measure the strength of the association between outcome variables and its determinant factors. Finally, *p* value < 0.05 declared a significant association.

### 2.7. Operational Definitions

#### 2.7.1. Stunting

Children are short for an age means that children have an HAZ of less than −2 SD on the WHO growth standard chart [16].

#### 2.7.2. Minimum Meal Frequency

Minimum is defined as the proportion of children aged 6–59 months, who receive solid, semisolid, or soft foods at the minimum numbers of two and three times for children aged 6–8 months and 9–59 months, respectively [15].

#### 2.7.3. Minimum Dietary Diversity Score

It is the proportion of infants and young children aged 6–59 months who received foods and drinks from 4 and more food groups in the previous 24 hrs. Consumption of any amount and quality of food from each food group was sufficient to “count” [15–17].

#### 2.7.4. Kebele

It has the smallest administrative structure with a household number of nearly 500.

## 3. Results

### 3.1. Sociodemographic Characteristics

A total of 767 participants were interviewed, yielding a response rate of 97.3%. The mean of the participant's mother in the completed year's age was 29 with a standard deviation of (±6.34) years, and 405 (52.8%) of them were aged between 25 and 34 years old. Majority, 721 (94.0%), of the mothers were married, and 511 (66.6%) were housewives. Regarding the educational status of the mother, 399 (52.0%) had an elementary school, while 178 (23.2%) had no formal education. Majority, 581 (75.7%), of the under-five-year aged children's family had more than 5 family members. In addition to this, more than one-third, 277 (36.1%), of them were from children whose fathers had a polygamous marriage. Three hundred five (42.4%) of under-five-year aged children's families were poor in wealth status. Regarding the household food security, 584 (76.1%) were mild/moderately insecure and 65 (8.5%) were severely insecure. Out of the under-five-year aged children participated, nearly half 392 (51.1%) were aged between 25 and 47 months and 389 (50.7%) were male (Table 1).

### 3.2. Healthcare and Other Related Characteristics

Out of 767 participants, 462 (60.2%) and 247 (32.2%) used piped water as a source of drinking water outside the compound or communal and inside their compound, respectively, while 34 (4.4%) and 20 (2.6%) still used protected spring and surface water, respectively. The majority of 728 (94.9%) had functional toilet facilities. Regarding the healthcare utilization-related factors, 144 (18.8%) of mothers had no antenatal care (ANC) follow-up and only 53 (6.9%) attended ANC4. In addition to this, 287 (37.4%) still had the experience of home delivery. Regarding the under-five-year aged children, the majority, 583 (76.0%), were vaccinated, and among these, 468 (80.3%) of them were fully vaccinated. Nearly one-third of 255 (33.2%) had diarrhea in the last two weeks and 138 (54.1%) sought modern healthcare. Most, 755 (98.4%), of them had breastfeeding, and 751 (97.9%) start breastfeeding with in the 1st one hour. The majority of 618 (80.6%) were breastfed for more than 18 months, while nearly half 368 (48.0%) of them started at 6 months of complementary feeding and 409 (54.9%) were fed by their hand (Table S1).

### 3.3. Dietary Diversity Score of Mothers

The mean dietary diversity score of food groups was 5.24 with a standard deviation (±SD) of ±1.41. The under-five-year aged children consumed the following: 736 (96.0%) consumed grains, white roots, and tubers, 442 (57.6%) consumed pulses such as beans, peas, and lentils, 614 (80.1%) consumed milk and milk product, and 738 (96.2%) consumed dark green leafy vegetables (Table S2).

### 3.4. The Prevalence of Stunting

**Figure 1:**
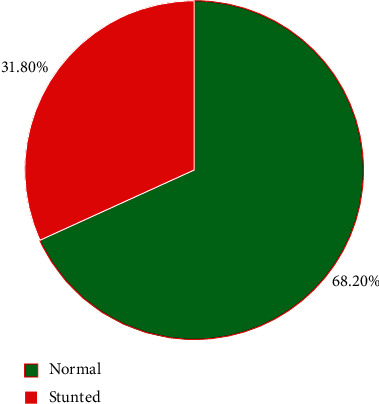
The prevalence of stunting among under-five children in the West Guji Zone, Oromia, Ethiopia, 2020.

The prevalence of stunting was 244 (31.8%) with 95% CI (28.6–35.2) among under-five-age children scored a z-score below −2 SD of the WHO standards or short for their age. Out of the study participants, 91 (11.9%) were severely stunted and 153 (19.9%) were moderately stunted (Figure 1). The prevalence of the stunting was varied, 101 (26.0%) vs. 143 (37.8%) among males and females, respectively.

### 3.5. Factors Affecting Stunting

The under-five children whose mother had no formal education and above 5 family members had a significant association with the bivariable logistic regression analysis, while others remain statistically significant after controlling for confounder at multivariable logistic regression. The under-five children whose fathers had a polygamous marriage were 3 times (AOR = 4.92, 95% CI: 3.46, 7.00) short for their age or stunted than them whose fathers had a monogamous marriage. Furthermore, a child being female sex (AOR = 1.74, 95% CI: 1.23, 2.47) was shorter than males for their age. A child who was not vaccinated (AOR = 1.75, 95% CI: 1.15, 2.67) was shorter than vaccinated children. A child who had less than four meal frequencies (AOR = 2.95, 95% CI: 1.56, 5.58), a child from poor households' wealth status (AOR = 3.03, 95% CI: 1.63, 5.63) and medium households' wealth status (AOR = 2.07, 95% CI: 1.10, 3.87), and a child from severely food insecure household (AOR = 2.92, 95% CI: 1.36, 6.24) were short for their age or stunted compared with their counterparts (Table 2).

## 4. Discussion

This community-based cross-sectional study revealed that the prevalence of stunting among under-five-age children was (31.8%) with 95% CI (28.6–35.2), and they scored a z-score below −2 SD or are short for their age. Out of the study participants, 11.9% were severely stunted and 19.9% were moderately stunted which is lower than the national prevalence of 37% of children under 5 who are short for their age or stunted (below −2 SD) from the 2019 EMDHS [11], 39.3% in Boricha Woreda/District, Southern Ethiopia [18], 47.9% in Arba Minch, Southern Ethiopia [13], 43.9% in Democratic Republic of Congo [19], 37% in Nepal [20], and 43.2% in Bangladesh [21]. This difference might be sociodemographic factors, low awareness, and other seasonal and cultural differences in ways of traditional food preparations among the study population [22]. This indicates that there are some improvements in the prevalence of stunting among under-five-age children which gives hope to attain the WHO and SDGs targets. This result higher than the previous study reported as follows: Hawassa Zuria District, Southern Ethiopia (26.6%) [23], Jima Zone, Southwest Ethiopia (24.1%) [24], Sodo Zuria District in South Ethiopia (24.9%) [12], the urban-rural gradient in Eastern Ethiopia (26.9%) [25], Adwa Town of North Ethiopia (12.2%) [26], and Brazil (29.9%) [20]. This can explain the variations between the different parts of the country, and this may be due to the different types of the interventions particularly by nongovernmental organizations and the agricultural activities, child-feeding practice, and the difference in socioeconomic activities. This may be due to a lack of nearby market access and lack of media access to get different advice about dietary diversity.

The study finding revealed that mothers who were not working currently, had any formal education, had more than 5 family size, and cannot feed their children minimum meal frequency had shorter children than their counterparts. This can be explained that the under-five children were most vulnerable to stunting due to low or poor family wealth status, low educational level, frequent round delivery or high family size, food insecurity of the household, and male sex preference of the household which were major determinants of the stunting among under-five children at the study area.

The study results revealed that under-five children whose fathers had a polygamous marriage were shorter for their age or stunted than them whose fathers had a monogamous marriage. This study result agreed with Kenya [27]. The polygamous marriage is commonly practiced in the study area and may favor a child to undernutrition and stunting among under-five children. In the case of the study area, most of the women are economically dependent on their husbands and they cannot afford the cost of well-nourished, minimum meal frequency, and dietary diversity on their own unless they are supported by their husbands. This implies that government bodies need to improve enforcement of the law for polygamy. It also needs better strategies to strengthen women's education and create job opportunities for women to reduce stunting among under-five children.

Furthermore, a child being female sex was shorter than males for their age, similarly reported from Rwanda and Sodo Zuria District, South Ethiopia [12]. This may be due to cultural issues, gender preference, and discrimination during the feed of their children. Despite having the above result, the study from Tigray Region, Northern Ethiopia, reported being a male sex child who was shorter than females for their age [28]. This difference may be due to the difference in socioeconomic and cultural acceptance of the gender in the community and maternal education, and the urban population may also have better exposure to mass media and have better information about gender equality.

A child who had less than four meal frequencies was more likely stunted than those who had more than four meal frequencies which agrees with Sodo Zuria District, South Ethiopia [12], and Northeast Ethiopia [29]. This may be due to that low food and nutrition intake leads to undernutrition and stunting. Health officials and clinicians need to emphasize pertinent health education on gender preference and discrimination during the feed of their children.

A child who was not vaccinated was shorter for their age or stunted compared with vaccinated children. This study result agreed with the previous study report from India [30]. It is also associated with infectious disease and diarrheal morbidity [31]. This might be preventive effect of the vaccine especially infectious disease and diarrhea which leads to malnutrition. Mothers who had not vaccinated their children also may be related with poor child care and feeding practice due to less access to mass media and to appropriate messages and campaigns on heath and vaccines. This needs better strategies to address mothers who had low income and had no formal education who live in shanty houses in the city.

A child from poor wealth status and severely food insecure households was shorter for their age or stunted compared with their counterparts, similarly reported from Arba Minch, Southern Ethiopia [13], Dabat, Ethiopia, and Boricha District, Southern Ethiopia [18]. This can be explained that children who were born in poor wealth status and severely food insecure household were facing a challenge to get food due to the number of the priorities to feed all under-five children and competition with elder children, and in low-income households, that may lead to low access to adequate dietary intake in kinds and the amounts. This implies that government bodies and other stakeholders should work together to improve the household wealth status and create job opportunities for women and maintain affirmative actions for women employment. It also needs better strategies to strengthen women's education and employment in the study area.

### 4.1. Limitations

There might be a potential for recall and social desirability bias on the child-feeding practice and socioeconomic. In addition to this, since the study is cross-sectional, it may not be strong to demonstrate a direct cause and effect relationship between risk factors and outcome. Since the study included children aged 24–59 months, there is a potential recall bias among respondents answering questions relating to events happening in the past two years and above, taking an extra meal during pregnancy or lactation and dietary diversity of children in the past 24 hrs.

## 5. Conclusions

This study result shows that nearly one-third of the under-five children are still short for their age or stunted. The under-five children whose fathers had a polygamous marriage, being a female sex child, had less than 4 meal frequencies/day, and being poor in wealth status and severely food insecure household were short for their age or stunted compared with their counterparts. It needs intervention on factors which attributes to stunting by providing health education on family planning and healthy child-feeding practices to combat or minimize gender-based discrimination among children at the community. In addition to this health officials in collaboration with other sectors, it is needed to act together to improve enforcement of the law for polygamous, the household's wealth status, and food security for the better health of a child and future. It also needs better strategies to strengthen women's education and employment to reduce stunting among under-five children.

## Figures and Tables

**Table 1:** Sociodemographic characteristics of respondents, West Guji Zone, Oromia, Ethiopia, 2020.

Category	No. (%)
*Age of the mother*	
18–24	191 (24.9)
25–34	405 (52.8)
35 or above	171 (22.3)

*Marital status*	
Unmarried	46 (6.0)
Married	721 (94.0)

*Occupation of mother*	
Housewife	511 (66.6)
Merchant	167 (21.8)
Employed and others*∗*	89 (11.6)

*Educational status of father*	
No formal education	178 (23.2)
Primary education	399 (52.0)
Secondary and above	190 (24.8)

*Maternal education*	
No formal education	280 (36.5)
Primary education	370 (48.2)
Secondary and above	117 (15.3)

*Family size*	
≤ 5	186 (24.3)
> 5	581 (75.7)

*Husband had polygamy*	
No	490 (63.9)
Yes	277 (36.1)

*Household's wealth*	
Wealthy	139 (18.1)
Medium	303 (39.5)
Poor	325 (42.4)

*Household food insecurity*	
Food secure	118 (15.4)
Mild/moderately insecure	584 (76.1)
Severely insecure	65 (8.5)

*Age of child*	
≤ 24	244 (31.8)
25–47	392 (51.1)
48–59	131 (17.1)

*Sex of child*	
Male	389 (50.7)
Female	378 (49.3)

*∗*Daily laborer and students.

**Table 2:** Bivariable and multivariable logistic regression analyses for stunting among under-five-age children in West Guji Zone, Oromia, Ethiopia, 2020.

	Height for age				
	Stunted (−2 SD) no. (%)	Normal no. (%)	COR (95% CI)	AOR (95% CI)	*P* value
*Maternal education*					
No formal education	108 (38.6)	172 (61.4)	2.00 (1.23, 3.25)	1.68 (0.96, 2.95)	0.068
Primary education	108 (29.2)	262 (70.8)	1.31 (0.81, 2.12)	1.09 (0.63, 1.89)	0.752
Secondary and above	28 (23.9)	89 (76.1)	1	1	

*Family size*					
≤5	43 (23.1)	143 (76.9)	1	1	
>5	201 (34.6)	380 (65.4)	1.76 (1.20, 2.58)	1.54 (0.99, 2.37)	0.051

*Husband had polygamy*					
No	96 (19.6)	394 (80.4)	1	1	
Yes	148 (53.4)	129 (46.6)	4.71 (3.40, 6.52)	4.92 (3.46, 7.00)	<0.001^*∗*^

*Household's wealth*					
Wealthy	17 (12.2)	122 (87.8)	1	1	
Medium	100 (33.0)	203 (67.0)	3.54 (2.02, 6.20)	2.07 (1.10, 3.87)	0.023^*∗*^
Poor	127 (39.1)	198 (60.9)	4.60 (2.62, 8.01)	3.03 (1.63, 5.63)	<0.001^*∗*^

*Sex of child*					
Male	101 (26.0)	288 (74.0)	1	1	
Female	143 (37.8)	235 (62.2)	1.74 (1.28, 2.36)	1.74 (1.23, 2.47)	0.002^*∗*^

*Meal frequency*					
≤ 4 meals per day	229 (35.5)	416 (64.5)	3.93 (2.23, 6.90)	2.95 (1.56, 5.58)	0.001^*∗*^
> 4 meals per day	15 (12.3)	107 (87.7)	1	1	

*Household food insecurity*					
Food secure	30 (25.4)	88 (74.6)	1	1	
Mild/Moderately insecure	189 (32.4)	395 (67.6)	1.40 (0.90, 2.20)	1.56 (0.93, 2.63)	0.091
Severely insecure	25 (38.5)	40 (61.5)	1.83 (0.96, 3.51)	2.92 (1.36, 6.24)	0.006^*∗*^

*Child vaccinated*					
Yes	45 (24.5)	139 (75.5)	1	1	
No	199 (34.1)	384 (65.9)	1.60 (1.10, 2.33)	1.75 (1.15, 2.67)	0.009^*∗*^

## Data Availability

The data used to support the findings are included within the article and available from the corresponding author upon request.

## Abbreviations

ANC: Antenatal care
AOR: Adjusted odds ratio
COR: Crude odds ratio
DDS: Dietary diversity score
EMDHS: Ethiopia Mini Demographic and Health Survey
ETB: Ethiopian birr
IYCF: Infant and young child feeding
SDGs: Sustainable Development Goals
SPSS: Statistical Package for Social Science
UNICEF: United Nations International Children's Emergency Fund
WHO: World Health Organization.

## Supplementary Materials

Supplementary Materials Supplementary Table S1: healthcare and environmental characteristics of respondents and Table S2: dietary diversity score of respondents, West Guji Zone, Oromia, Ethiopia, 2020. Supplementary File 2: English questionnaires and assessment tools used to assess prevalence of stunting and associated factors among under-five-age children.
