# Supplementary material for: Stunting and Associated Factors among Under-Five-Age Children in West Guji Zone, Oromia, Ethiopia
Source: J Nutr Metab. 2021 Feb 4;2021:8890725. doi: 10.1155/2021/8890725 (PMC7878078; doi:10.1155/2021/8890725)
Supplement: Supplementary Materials — Supplementary Table S1: healthcare and environmental characteristics of respondents and Table S2: dietary diversity score of respondents, West Guji Zone, Oromia, Ethiopia, 2020. Supplementary File 2: English questionnaires and assessment tools used to assess prevalence of stunting and associated factors among under-five-age children. [file 8890725.f1.zip › 8890725.f1/Supplementary file 1 -Table S1-2 (1).docx]

Table S1: Health care and Environmental characteristics of respondents, West Guji zone, Oromia, Ethiopia, 2020.

| **Variables** | **Category** | **No.** | **(%)** |
| --- | --- | --- | --- |
| Source of Drinking water | Piped outside the compound | 462 | (60.2) |
|  | Piped in to compound | 247 | (32.2) |
|  | Tube well or borehole | 4 | (.5) |
|  | Protected spring | 34 | (4.4) |
|  | surface water | 20 | (2.6) |
| Functional toilet facility | No | 39 | (5.1) |
|  | Yes | 728 | (94.9) |
| ANC visits | None | 144 | (18.8) |
|  | 1 | 40 | (5.2) |
|  | 2-3 | 530 | (69.1) |
|  | 4+ | 53 | (6.9) |
| Place of delivery | Public health facility | 477 | (62.2) |
|  | Private health Facility | 3 | (.4) |
|  | Home | 287 | (37.4) |
| Child vaccinated | Yes | 583 | (76.0) |
|  | No | 184 | (24.0) |
| Vaccination status | fully vaccinated | 468 | (80.3) |
|  | up to date for the child’s age | 113 | (19.4) |
|  | not fully vaccinated | 2 | (.3) |
| Diarrhea | Yes | 255 | (33.2) |
|  | No | 512 | (66.8) |
| Seek for health care | Yes | 138 | (54.1) |
|  | No | 117 | (45.9) |
| Ever breast fed | Yes | 755 | (98.4) |
|  | No | 12 | (1.6) |
| when did breastfeeding started | <= 1 hour of birth | 751 | (97.9) |
|  | within the first hour | 13 | (1.7) |
|  | >24hours | 3 | (.4) |
| Colostrum | Yes | 649 | (84.6) |
|  | No | 118 | (15.4) |
| any foods or fluids | Yes | 262 | (34.2) |
|  | No | 505 | (65.8) |
| Breast fed for more than 18 months | No | 149 | (19.4) |
|  | Yes | 618 | (80.6) |
| Complementary feeding started at 6 months | No | 399 | (52.0) |
|  | Yes | 368 | (48.0) |
| Hand | No | 336 | (45.1) |
|  | Yes | 409 | (54.9) |
| Bottle | No | 621 | (83.4) |
|  | Yes | 124 | (16.6) |

Table S2: Dietary diversity score of respondents, West Guji zone, Oromia, Ethiopia, 2020.

| **Food groups** (n=767) | **Ever had** | | **Daily** | | **1-3 per weak** | | **1-3 per month** | | **Not/once/year** | |
| --- | --- | --- | --- | --- | --- | --- | --- | --- | --- | --- |
|  | **Freq.** | **(%)** | **Freq.** | **(%)** | **Freq.** | **(%)** | **Freq.** | **(%)** | **Freq.** | **(%)** |
| Grains white roots tubers | 736 | (96.0) | 421 | (95.2) | 21 | (4.8) | 0 | (.0) | 0 | (.0) |
| Pulses beans peas and lentils | 442 | (57.6) | 71 | (58.7) | 36 | (29.8) | 14 | (11.6) | 0 | (.0) |
| Nuts and seeds | 121 | (15.8) | 0 | (.0) | 46 | (7.5) | 113 | (18.4) | 455 | (74.1) |
| Milk and milk product | 614 | (80.1) | 371 | (48.4) | 387 | (50.5) | 9 | (1.2) | 0 | (.0) |
| Meat poultry fish | 138 | (18.0) | 15 | (10.9) | 85 | (61.6) | 16 | (11.6) | 22 | (15.9) |
| Eggs | 152 | (19.8) | 88 | (57.9) | 22 | (14.5) | 17 | (11.2) | 23 | (15.1) |
| Dark green leafy vegetables | 738 | (96.2) | 658 | (89.2) | 80 | (10.8) | 0 | (.0) | 0 | (.0) |
| Vit A rich fruits and vegetable | 299 | (39.0) | 32 | (4.2) | 319 | (41.6) | 339 | (44.2) | 77 | (10.0) |
| Other vegetables | 527 | (68.7) | 656 | (85.5) | 111 | (14.5) | 0 | (.0) | 0 | (.0) |
| Other fruits | 250 | (32.6) | 265 | (78.9) | 25 | (7.4) | 18 | (5.4) | 28 | (8.3) |
