# Supplementary material for: Stunting and Associated Factors among Under-Five-Age Children in West Guji Zone, Oromia, Ethiopia
Source: J Nutr Metab. 2021 Feb 4;2021:8890725. doi: 10.1155/2021/8890725 (PMC7878078; doi:10.1155/2021/8890725)
Supplement: Supplementary Materials — Supplementary Table S1: healthcare and environmental characteristics of respondents and Table S2: dietary diversity score of respondents, West Guji Zone, Oromia, Ethiopia, 2020. Supplementary File 2: English questionnaires and assessment tools used to assess prevalence of stunting and associated factors among under-five-age children. [file 8890725.f1.zip › 8890725.f1/Supplementary file 2 -English Questionnaires.doc]

## Annex III English Questionnaires

Our study focuses to assess prevalence of stunting and associated factors among under five age children in West Guji Zone, Oromia, Ethiopia: A Community-Based Cross-Sectional Study

Name of the data collector__________________ signature_________ Data________

Questionnaire code_____________________

**Part I: Socio- demographic and economic characteristics of households**

| **S.No** | **Questions** | **Response and coding category** | **Skip** |
| --- | --- | --- | --- |
| 1 | Characteristics of the child |  |  |
| 101 | How many months old is your child? | …………………….(months) |  |
| 102 | What is the sex of your child? | 1. Male 2. Female |  |
| 103 | What is the birth order your child? | 1. 1 2. 2-3 3. 4-5 4. 6+ |  |
| 104 | How many months does your child have between the preceding birth interval | 1. First birth 2. <24 months 3. ≥24 months |  |

**Part II. Characteristics of the parent and Household**

| **S.No** | **Questions** | **Response and coding category** | **Skip** |
| --- | --- | --- | --- |
| 201 | How old are you? | …………………. (yrs.) |  |
| 202 | What is your marital status? (Mother) | 1. Married 2. Divorced 3. Widowed 4. Never married |  |
| 203 | Did your husband had polygamy? | 1. Yes 2. No |  |
| 204 | What is your ethnicity? | 1. Guji 2. Gedeo 3. Amhara 4. Guraghe 5. Sidama 6. Silte 7. Other specify? ________ |  |
| 205 | What is your religion? | 1. Protestant 2. Orthodox 3. Muslim 4. Catholic 5. Others (specify) ………… |  |
| 206 | What is your main occupation? (Mother) | 1. House wife 2. Government employee 3. Non-government employee 4. Merchant 5. Self-employee 6. Others (specify) ……… |  |
| 207 | What is your husband’s main occupation? | 1. Government employee 2. Non-Government employee 3. Merchant 4. Self-employee 5. Others (specify)…... |  |
| 208 | What is your educational | 1. No education 2. Primary education (1-8) 3. Secondary (9-12) 4. Above secondary |  |
| 209 | What is your husband’s educational level? | 1. No education 2. Primary education (1-8) 3. Secondary (9-12) 4. Above secondary |  |
| 210 | How much is your monthly income in birr? | ………….. birr |  |

**Part III. Household assets**

| **Sr.NO** | **Questions** | **Responses** | **Remark** |
| --- | --- | --- | --- |
| 301 | Do you have any of the following? (more than one response is possible) | 1. TV 2. Radio 3. Electricity 4. Cell phone/ Mobile 5. Table 6. Chair |  |
| 302 | What kind of toilet facility do you use? | 1. Pour flush latrine 2. Ventilated Improved pit 3. Pit latrine with slab 4. Open pit 5. No latrine |  |
| 303 | Do you have a livestock in your household? | 1. Yes 2. No |  |
| 304 | What are livestock holdings in your house? (>1 answer is possible) | 1. Oxen 2. Cows 3. Sheep 4. Goat 5. Chicken 6. Mules 7. Donkey 8. Hourses |  |
| 305 | Does the household have own land that can be used for agriculture? | 1. Yes 2. No | If No, Skip to Q 401 |
| 306 | What is the size (local unit) of agriculture land? | _______________hectar |  |

**Part IV. Environmental factors**

| **S.No** | **Questions** | **Response and coding category** | **Skip** |
| --- | --- | --- | --- |
| 401 | What is the main source of drinking water for members of your household? | 1. Piped in to compund 2. Piped outside compound 3. Tube well or borehole 4. Unprotected well 5. Protected spring 6. Surface water (river/dam/lake/pond/stream) 7. Other, specify ________ |  |
| 402 | Do you have functional toilet facility in our house? (if possible observe it) | 1. Yes  2. No |  |

**Part V. Health care factors**

| **S.No** | **Questions** | **Response and coding category** | **Skip** |
| --- | --- | --- | --- |
| 501 | How many times you visited Antenatal care in health institution when you are pregnant of this child? | 1. none 2. 1 3. 2-3 4. 4^+^ 5. don^′^t know |  |
| 502 | Where did you given birth your child? | 1. Public health facility 2. Private health facility 3. Home |  |
| 503 | Did you attend post-natal care service after delivery of your child? | 1. Yes 2. No |  |
| 504 | How much is your child’s weight or size at birth? | 1. Smaller than average   (<2.5kg)   2. Average (2.5 -4 kg) 3. Large (>4kg) |  |
| 505 | Did your child received vaccination? | 1. Yes 2. No | If no skip to 507 |
| 506 | If yes did your child took all  Vaccination? check if the card is available | 1. Fully Vaccinated 2. Up to date for the age of the child 3. Not-fully vaccinated |  |
| 507 | Did your child have been ill with diarrhea in the last two weeks | 1. Yes 2. No | If No, Skip to Q 601 |
| 508 | Did you child have recurrent episodes of diarrhea (2times and more) in the past two weeks? | 1. Yes 2. No |  |

**Part VI. Dietary factors**

| **S.No** | **Questions** | **Response and coding category** | **Skip** |
| --- | --- | --- | --- |
| 601 | Have you ever breast fed your child? | 1. Yes 2. No | If no skip to 606 |
| 602 | When did you start breast feeding after delivery? | 1. ≤1 hour of birth 2. Within the first 24 hours 3. >24 hours |  |
| 603 | Did your child receive the first milk (colostrums)? | 1. Yes 2. No |  |
| 604 | Did your child receive any food or liquids other than breast milk during the first three days of life? | 1. Yes 2. No |  |
| 605 | For how long the child breast fed? | ……………….. (months) |  |
| 606 | When did you start giving complementary foods to your child? | ……………….. (months) |  |
| 607 | What method did you used for feeding your child? | 1. Spoon 2. Cup 3. Hand 4. bottle | More than one answer is possible |
|  | Dietary Diversity (ask the care taker what the child have eaten in the previous 24hours) |  |  |
| 608 | Grain, roots or tubers? (Bread, rice, pasta, maize, teff, sweet potato, potato, cassava, garlic, onion etc.) | 1. Yes 2. No |  |
| 609 | legumes and nuts (beans, peas, chicken peas, lentils, sunflower etc.) | 1. Yes 2. No |  |
| 610 | dairy products (milk, yogurt, cheese, *arera*) | 1. Yes 2. No |  |
| 611 | flesh foods (meat, fish, poultry and liver/organ meats) | 1. Yes 2. No |  |
| 612 | Eggs | 1. Yes 2. No |  |
| 613 | vitamin-A rich fruits and vegetables (carrots, mango, papaya *etc.)* | 1) Yes 2) No |  |
| 614 | Other fruits and vegetables (Cabbages, Avocado, Banana, Orange, tomato, mushrooms, pumpkin etc.) | 1. Yes 2. No |  |
| 615 | How many times did the child fed? (include snacks if any) | ____________ in number times the child fed |  |

**Part VII. Household food insecurity (these questions should be asked for the period of the previous four weeks)**

| Sr.NO | Questions | Response | Remark |
| --- | --- | --- | --- |
| 701 | Did you worry that your household would not have enough food? | 1. Yes 2. No | If No, skip to 702 |
| 701a | How often did this happen? | 1. Rarely (1 or 2 times) 2. Sometimes (3 to 10 times) 3. Often (more than 10 times) |  |
| 702 | Were you or any household member not able to eat the kinds of foods you preferred because of a lack of resources? | 1) Yes 2) No | If No, skip to 703 |
| 702a | How often did this happen? | 1. Rarely (1 or 2 times) 2. Sometimes (3 to 10 times) 3. Often (more than 10 times) |  |
| 703 | Did you or any household member have to eat a limited variety of foods due to a lack of resources? | 1. Yes 2. No | If No, skip to 704 |
| 703a | How often did this happen? | 1. Rarely (1 or 2 times) 2. Sometimes (3 to 10 times) 3. Often (more than 10 times) |  |
| 704 | Did you or any household member have to eat some foods that you really did not want to eat because of a lack of resources to obtain other types of food? | 1. Yes 2. No | If No, skip to 705 |
| 704a | How often did this happen? | 1. Rarely (1 or 2 times) 2. Sometimes (3 to 10 times) 3. Often (more than 10 times) |  |
| 705 | Did you or any household member have to eat a smaller meal than you felt you needed because there was not enough food? | 1. Yes 2. No | If No, skip to 706 |
| 705a | How often did this happen? | 1. Rarely (1 or 2 times) 2. Sometimes (3 to 10 times) 3. Often (more than 10 times) |  |
| 706 | Did you or any other household member have eaten fewer meals in a day because there was not enough food? | 1. Yes 2. No | If No, skip to 707 |
| 706a | How often did this happen? | 1. Rarely (1 or 2 times) 2. Sometimes (3 to 10 times) 3. Often (more than 10 times) |  |
| 707 | Was there ever no food to eat of any kind in your household because of lack of resources to get food? | 1. Yes 2. No | If No, skip to 708 |
| 707a | How often did this happen? | 1. Rarely (1 or 2 times) 2. Sometimes (3 to 10 times) 3. Often (more than 10 times) |  |
| 708 | Did you or any household member go to sleep at night hungry because there was not enough food? | 1. Yes 2. No | If No, skip to 709 |
| 708a | How often did this happen? | 1. Rarely (1 or 2 times) 2. Sometimes (3 - 10 times) 3. Often (> 10 times) |  |
| 709 | Did you or any household member go a whole day and night without eating anything because there was not enough food? | 1. Yes 2. No | If No, skip to 8 |
| 709a | How often did this happen? | 1. Rarely (1 or 2 times) 2. Sometimes (3 to 10 times) 3. Often (more than 10 times) |  |

**Part VIII. Anthropometry**

| Sr.NO | Anthropometric measurement | Measurement in cm | Remark |
| --- | --- | --- | --- |
| 8 | Height measurement |  |  |
| 801 | Height | ………………. (cm) |  |

**Thank you for your participation!!**
